# Cellular responses to T-2 toxin and/or deoxynivalenol that induce cartilage damage are not specific to chondrocytes

**DOI:** 10.1038/s41598-017-02568-5

**Published:** 2017-05-22

**Authors:** Yang Lei, Zhao Guanghui, Wang Xi, Wang Yingting, Lin Xialu, Yu Fangfang, Mary B. Goldring, Guo Xiong, Mikko J. Lammi

**Affiliations:** 1 School of Public Health, Health Science Center, Xi’an Jiaotong University, Key Laboratory of Trace Elements and Endemic Diseases of National Health and Family Planning Commission, Xi’an, Shaanxi P.R. China; 2 Hong Hui Hospital, Health Science Center, Xi’an Jiaotong University, Xi’an, Shaanxi P.R. China; 3 Hospital for Special Surgery, Weill Cornell Medical College, New York, USA; 4 Department of Integrative Medical Biology, University of Umeå, Umeå, Sweden

## Abstract

The relationship between T-2 toxin and deoxynivalenol (DON) and the risk of Kashin-Beck disease is still controversial since it is poorly known about their selectivity in cartilage damage. We aimed to compare the cytotoxicity of T-2 toxin and DON on cell lines representative of cell types encountered *in vivo*, including human chondrocytes (C28/I2), human hepatic epithelial cells (L-02) and human tubular epithelial cells (HK-2). In addition, we determined the distribution of T-2 toxin and DON in Sprague-Dawley (SD) rats after a single dose exposure. T-2 toxin or DON decreased proliferation in a time- and concentration-dependent manner and their combination showed a similar antagonistic effect in C28/I2, L-02 and HK-2 cells. Moreover, we observed cell cycle arrest and apoptosis, associated with increased oxidative stress and decline in mitochondrial membrane potential induced by T-2 toxin and/or DON. *In vivo* study showed that T-2 toxin and DON did not accumulate preferentially in the knee joint compared to liver and kidney after an acute exposure in SD rats. These results suggest that T-2 toxin and/or DON inhibit proliferation and induce apoptosis through a possible mechanism involving reactive oxygen species-mediated mitochondrial pathway that is not specific for chondrocytes *in vitro* or joint tissues *in vivo*.

## Introduction

The trichothecenes are a large group of secondary metabolites mainly produced by the fungi of Fusarium genus^1^. They are commonly found on various cereals, including maize, wheat and barley, grown in the temperate regions of the world^2^. Ingestion of these toxins via contaminated food and feed can cause serious adverse effects on humans and animals^3^. The most prominent toxic effect of trichothecenes on eukaryotic cells is the inhibition of protein and nucleic acid synthesis, as well as induction of cell apoptosis^4, 5^. The T-2 toxin and deoxynivalenol (DON), belonging to type A and type B trichothecenes, are highly toxic and the most common mycotoxins because of their widespread dissemination^1^. Both T-2 toxin and DON promote the generation of cellular reactive oxygen species (ROS), which can further induce lipid peroxidation, DNA damage, and cell apoptosis in different cell types^6, 7^. Due to their co-occurrence in nature, the combined toxic effects of these mycotoxin mixtures have received more consideration^8–12^.

Kashin-Beck disease (KBD) is a chronic, disabling osteoarticular disease mainly distributed in a limited endemic area from Northeast to Southwest China, Southeastern Siberia, and North Korea^13, 14^. The onset of KBD commonly occurs in preadolescent and adolescent years, and leads to various types of disabilities in adult life^15^. According to the China statistical yearbook of health and family planning in 2015, there were 0.61 million patients with KBD, of which 13,453 children were under 13 years of age in 378 counties of China^16^. The incidence of KBD is variable, so that 60–90% of children may reveal signs of KBD in certain seriously affected regions^17^. In general, KBD incidence has been markedly reduced in recent years, but new patients are still detected in the western regions of China, particularly in the Qinghai and Sichuan provinces and Tibet Autonomous Regions^18^. The commonly affected sites of KBD patients are the epiphyseal growth plate and the articular cartilage with pathology characterized by chondronecrosis, which can result in impaired endochondral ossification, growth retardation, and secondary chronic osteoarthropathy^14^. In addition, oxidative stress^19^, mitochondrial dysfunction^20^ and excessive apoptosis of chondrocytes^21^ contribute to the pathology of KBD. Clinically, the disease manifests in enlarged and shortened fingers, arthritic pain, morning stiffness, and deformed, enlarged joints with limited motion in the extremities^22^.

The etiology of KBD remains elusive, although many hypotheses have been proposed. Contamination of food by mycotoxins, especially T-2 toxin and DON have been widely considered as risk factor for KBD mainly based on extensive epidemiological studies, which showed that cereal or food samples analyzed from KBD areas were more heavily contaminated with these toxins compared to those in non-KBD areas^23–28^. Changing grains has had obvious effects in the prevention and treatment of KBD in children^29^. In addition to these observations, *in vivo* and *in vitro* studies have also been done to show possible relationships between these mycotoxins and KBD occurrence^30–32^. Because no obvious damage was observed in anatomical sites in KBD patients other than the epiphyseal growth plate and articular cartilage, it appeared that the risk factors for KBD were selective for cartilage. However, previous studies only explored the toxic effects of mycotoxins on cartilage but without analysis of other tissues or organs as controls, and no study explored the effects of combinations of mycotoxins on cartilage.

Thus, to verify if different mycotoxins, either alone or in combination, could specifically damage cartilage, and to obtain more evidence about the relationship between mycotoxins and KBD prevalence, we compared the potential of T-2 toxin and/or DON to cause toxicity in cartilage, as well as hepatotoxicity and renal toxicity by using cell lines derived from those tissue. Thus, we assessed human chondrocyte (C28/I2), human hepatic epithelial cells (L-02), and human tubular epithelial cells (HK-2) for changes in cell viability, morphological changes, cell cycle progression and apoptosis, as well as oxidative stress (ROS) and mitochondrial membrane potential in response to T-2 toxin and DON alone and together. In addition, to verify if mycotoxins could accumulate in joint tissues, we examined their tissue distribution in knee joint, liver and kidney of Sprague-Dawley (SD) rats after acute oral administration of T-2 toxin and DON.

## Materials and Methods

### Chemicals

T-2 toxin solution (NO. 34071, 100 μg/ml in acetonitrile), DON solution (NO. 34124, 100 μg/ml in acetonitrile) and 3-[4,5-Dimethylthiazol-2-yl]-2,5- diphenyltetrazolium bromide (MTT) were purchased from Sigma Chemical Co. (St. Louis, MO, USA). Dulbecco’s modified Eagle’s medium-F12 (DMEM-F12) and fetal bovine serum (FBS) were purchased from Gibco BRL (Grand Island, NY, USA). All other chemicals used were of analytical grade.

### Cell culture and treatment

The human chondrocytic cell line (C28/I2) was reported previously^33^. The human hepatic epithelial cell line (L-02) and human tubular epithelial cell line (HK-2) were purchased from CHI Scientific Inc. (Jiangsu, China). All the three cell lines were maintained in monolayer cultures in DMEM-F12 containing 10% FBS, 100 IU/ml penicillin and 100 μg/ml streptomycin at 37 °C in a humidified atmosphere of 5% CO_2_ in air.

The working solutions of T-2 toxin and DON were made by direct dilution in culture medium. In each experiment, cells were plated and incubated for 24 h to allow them to attach before treatment with mycotoxins, and untreated cells were used as controls. All studies were tested in three independent experiments.

### Determination of cytotoxicity

The MTT assay was used to determine the cytotoxicity after exposure to T-2 toxin and/or DON. Briefly, cells in 96-well plates were treated with five dilutions of T-2 toxin (from 2.5 to 40 ng/ml) or DON (from 100 to 1600 ng/ml), or a fixed constant ratio of the two mycotoxins (T-2 + DON, ratio = 1:40)^11^. Following 24, 48 and 72 h of exposure, the medium was replaced by fresh medium containing 20 μl (5 mg/ml PBS) MTT. After 4 h of incubation, the medium was removed and 150 μl DMSO was added to dissolve the formazan. Measurement of the absorbance was performed with an automatic ELISA reader (Infinite M200, Tecan, Switzerland) at 490 nm.

The combined effects of T-2 toxin and DON mixtures were analyzed using an isobologram method^34, 35^. According to the isobologram analysis, a combination index (CI) value was calculated for quantification of synergism or antagonism for two drugs, as below:

1 $${\rm{CI}}=\frac{{(D)}_{1}}{{({D}_{x})}_{1}}+\frac{{(D)}_{2}}{{({D}_{x})}_{2}}=\frac{{(D)}_{1}}{{({D}_{m})}_{1}{[{f}_{a}/(1-{f}_{a})]}^{1/{m}_{1}}}+\frac{{(D)}_{2}}{{({D}_{m})}_{2}{[{f}_{a}/(1-{f}_{a})]}^{1/{m}_{2}}}$$

where D is the dose (or concentration) of a drug, D_m_ is the median-effect dose (e.g., IC50, ED50, or LD50) that inhibits the system under study by 50%, f_a_ is the fraction affected by D (e.g., percentage inhibition/100), and m is the coefficient signifying the shape of the dose-effect relationship. Dm and m values are used for calculating the CI values, where CI < 1, CI = 1, and CI > 1 indicate synergistic, additive, and antagonistic effects, respectively^35^. The calculations were achieved by the CalcuSyn software (Biosoft, Cambridge, UK).

### Observation of cell ultrastructure changes

Based on the IC30 values of 10.42 ng/ml for T-2 toxin and 842.17 ng/ml for DON after 24-h-exposure of C28/I2 cells (calculated by CalcuSyn software), we used 10 ng/ml of T-2 toxin and 800 ng/ml of DON, alone or in combination as the intervention concentrations in the subsequent experiments.

Cells were treated with T-2 toxin and/or DON in 6-well plates for 24 h, then were collected by digestion with trypsin, and fixed with 2.5% glutaraldehyde, post-fixed in 0.1% osmium tetroxide and embedded in Epon epoxy resin. Ultrathin sections were cut, stained with 0.1% lead citrate and 10% uranyl acetate. The ultrastructure of cells was observed with a transmission electron microscope (HITACHI H-7650, Tokyo, Japan).

### Hoechst 33324 staining

The morphological alterations of nuclei associated with apoptosis were observed with a Hoechst 33224 staining kit from Sigma Chemical Co. (St. Louis, MO, USA). Briefly, the cells were cultured in 6-well plates and exposed to T-2 toxin (10 ng/ml) or DON (800 ng/ml), alone or in combination for 24 h, the medium was removed, and cells were washed twice with PBS. Following incubation of the cells with 10 μg/ml Hoechst 33342 dye for 20 min at 37 °C, fragmented and intact nuclei were observed with an inverted fluorescence microscope (Nikon, Tokyo, Japan).

### Flow cytometry analysis of cell cycle and apoptosis

The cell cycle was determined using a cell cycle assay kit (CWBIO, Beijing, China) according to the manufacturer’s instructions. Following treatment with T-2 toxin (10 ng/ml) or DON (800 ng/ml), alone or in combination, in 6-well plates for 24 h, the cells were collected and washed once with cold PBS and fixed in 95% cold ethanol for 2 h. Then the fixed cells were washed once with PBS, treated with 400 μl propidium iodide (PI) dye solution, and incubated at 37 °C in the dark for 30 min before measurement.

An Annexin V-FITC/PI detection kit (4 A Biotech Co., Ltd, Beijing, China) was used to determine the cell apoptosis rates following the treatment of mycotoxins, as described above. Briefly, the cells were harvested, washed with cold PBS twice, and resuspended in 100 μl of binding buffer. After addition of 5 μl of Annexin V-FITC and incubation for 5 min in the dark, 10 μl of 20 mg/ml PI dye solution was added followed by addition of 400 μl of PBS for measurement.

Flow cytometric analysis (FACS) of cell cycle progression and apoptosis rates were performed using a Facscalibur Flow Cytometer (Becton Dickinson, Mountain View, CA, USA). Cell cycle and apoptosis data were acquired with the CellQuest software (BD Biosciences).

### Measurement of ROS

The oxidative stress level was assessed by measurement of ROS with a fluorescent dye DCFH-DA assay kit (Beyotime, Jiangsu, China). In brief, cells were cultured in 6-well plates and exposed to T-2 toxin (10 ng/ml) or DON (800 ng/ml), alone and in combination for 24 h. DCFH-DA was added and cells were incubated at 37 °C for 30 min. Then, the cells were washed three times with serum-free medium and visualized by an inverted fluorescence microscope (Nikon, Tokyo, Japan). The fluorescence intensity was analyzed by Image-Pro Plus 6.0 software.

### Mitochondrial membrane potential assay

Mitochondrial transmembrane potential (ΔΨm) was measured using a fluorescent dye JC-1 assay kit (Beyotime, Jiangsu, China). Following treatment with T-2 toxin (10 ng/ml) or DON (800 ng/ml), alone or in combination in 6-well plates for 24 h, cells were collected and resuspended in 0.5 ml of fresh medium. After addition of 0.5 ml JC-1 work solution, the cells were incubated at 37 °C for 20 min. Then, cells were washed twice and re-suspended in JC-1 buffer solution. The fluorescence was measured using a Facscalibur Flow Cytometer (Becton Dickinson, Mountain View, CA, USA). Data were processed with the CellQuest software (BD Biosciences).

### Experimental animals and groups

All animal protocols were approved by the Animal Ethics Committee of Xi’an Jiaotong University and were performed in accordance with the Guide for the Care and Use of Laboratory Animals of the National Institutes of Health. Eighteen 3-week-old SD rats weighing 30~60 g were purchased from Animal Experimental Centre of Xi’an Jiaotong University and randomly divided into 3 groups. Group A (6 rats) were administered with T-2 toxin as a single dose at 2 mg/kg body weight (bw) by oral gavage. Group B (6 rats) were administered with DON as a single dose at 10 mg/kg bw by oral gavage. Group C (6 rats) were given double distilled water at 0.2 ml/kg bw by oral gavage, and they were used to verify the viability of the analytical method by recovery tests. After 8 h of exposure, rats were euthanized, and the liver, kidney, and knee joint were collected. The samples were stored at −20 °C prior to their analysis.

### Measurement of T-2 toxin and DON by ELISA

The T-2 toxin and DON concentrations in tissues were analyzed using a AgraQuant^®^ T-2 toxin ELISA kit (COKAQ6000, Romer Labs Singapore Pte Ltd., Singapore) and a RIDASCREEN^®^ DON ELISA kit (R5906, R-Biopharm, Darmstadt, Germany), respectively. The tissue homogenates were prepared, as described by Pestka *et al*.^36^. The assays were performed according to the manufacturer’s protocols. After the reaction was stopped, the absorbance was measured with an automatic ELISA reader (Infinite M200, Tecan, Switzerland) at 450 nm for both T-2 toxin and DON. The concentrations of T-2 toxin and DON were quantified according to a standard curve. Data were reported as T-2 toxin or DON equivalents per g of organ tissue.

### Statistical analyses

Data were expressed as mean ± standard error (SEM) or standard deviation (SD), as calculated by SPSS software. Statistical analyses were performed by one-way ANOVA among groups, and the Student’s t test was employed to determine the significant differences between two groups. P < 0.05 was considered to be significant.

## Results

### Cytotoxicity of mycotoxins T-2 toxin and/or DON

**Figure 1:**
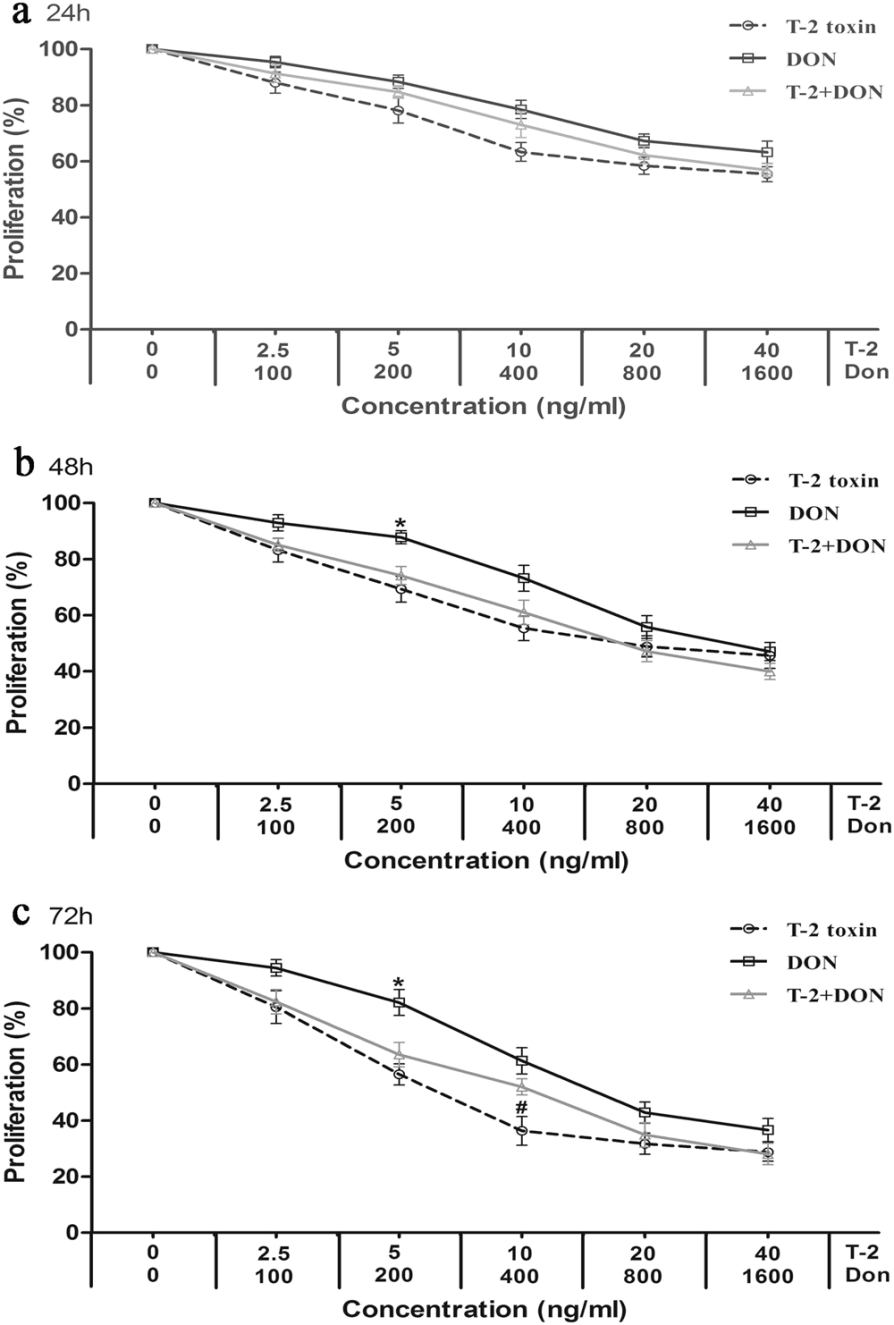
Cytotoxicity of T-2 toxin and/or DON in the cartilage-derived cell line, C28/I2. Cells in 96-well plates were exposed to five dilutions of T-2 toxin (from 2.5 to 40 ng/ml) or DON (from 100to 1600 ng/ml), or a fixed constant ratio of the two mycotoxins (T-2 + DON, ratio = 1:40). After (**a**) 24 h, (**b**) 48 h and (**c**) 72 h of exposure, the cell viability was analyzed by the MTT assay. Each data point represents the mean ± SEM from three independent experiments with replicate samples, *p < 0.05 indicates significant differences between the mixture and T-2 toxin alone, ^#^p < 0.05 represents significant differences between the mixture and DON alone.

**Figure 2:**
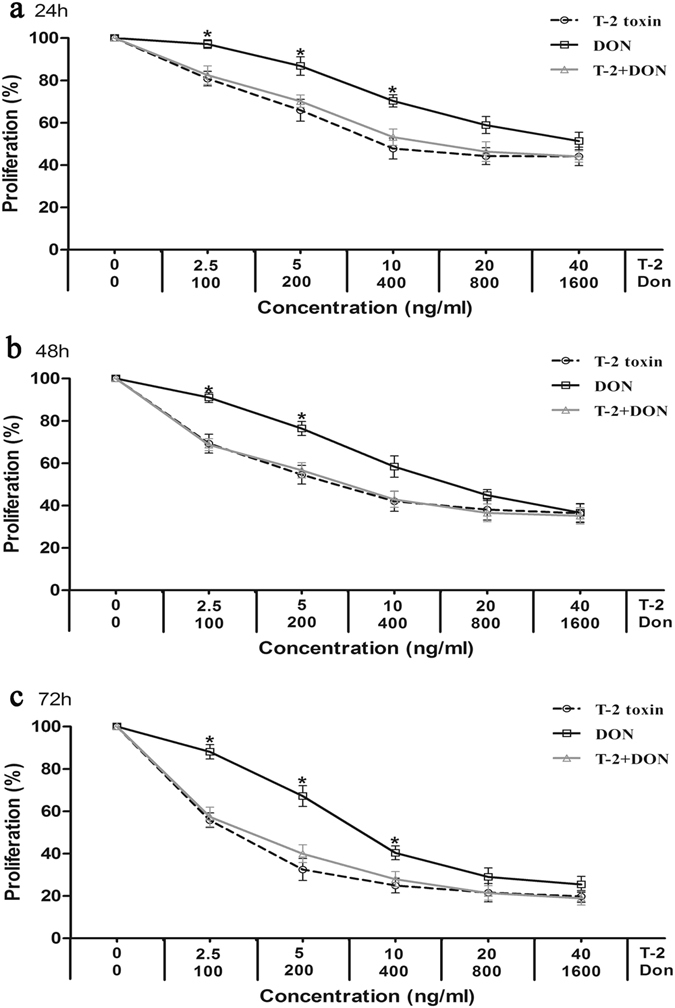
Cytotoxicity T-2 toxin and/or DON in the liver-derived cell line, L-02. Cells were exposed to mycotoxins for (**a**) 24 h, (**b**) 48 h and (**c**) 72 h and analyzed by the MTT method, as described in Fig. 1. Each data point represents the mean ± SEM from three independent experiments with replicate samples, *p < 0.05 indicates significant differences between the mixture and T-2 toxin alone, ^#^p < 0.05 represents significant differences between the mixture and DON alone.

**Figure 3:**
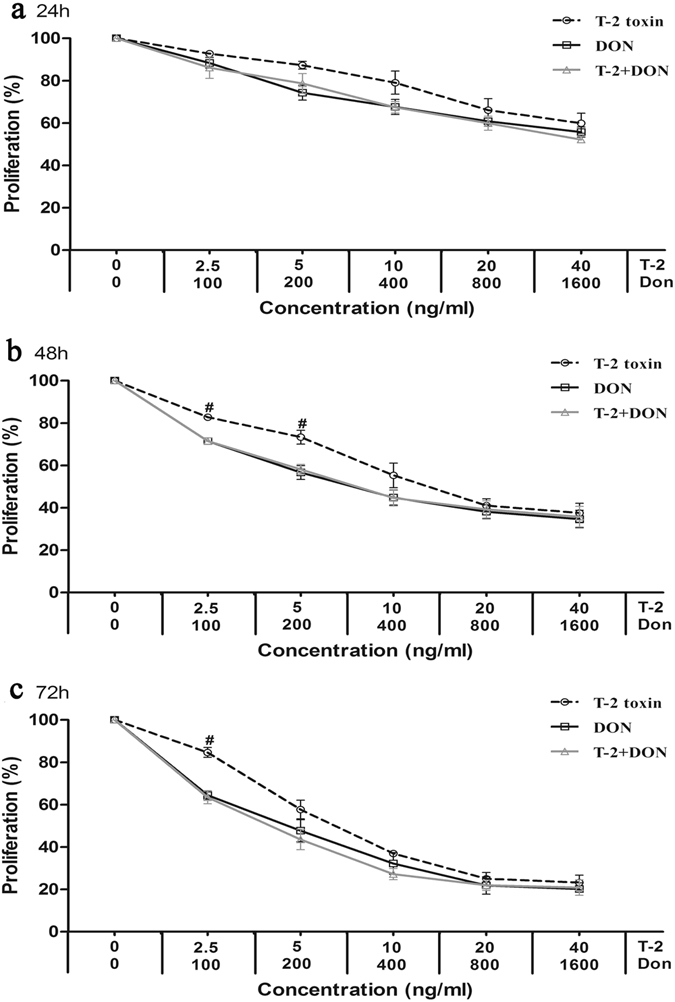
Cytotoxicity of T-2 toxin and/or DON in the kidney-derived cell line, HK-2. Cells were exposed to mycotoxins for (**a**) 24 h, (**b**) 48 h and (**c**) 72 h and analyzed by the MTT method, as described in Fig. 1. Each data point represents the mean ± SEM from three independent experiments with replicate samples, *p < 0.05 indicates significant differences between the mixture and T-2 toxin alone, ^#^p < 0.05 represents significant differences between the mixture and DON alone.

The C28/I2, L-02 and HK-2 cells were evaluated for cell viability by MTT assay after exposure to T-2 toxin or DON alone or in combination. As shown in Figs 1, 2, and 3, cell proliferation, assessed as % of untreated control set at 100%, decreased in a time- and concentration-dependent manner in all three cell lines.

The relative cytotoxic effects were evaluated by calculating the concentration of mycotoxin that produced 50% inhibition of cellular proliferation (IC50). The IC50 values of both mycotoxins decreased with the increased exposure time (Table 1). According to the IC50 values, T-2 toxin was less cytotoxic on C28/I2 and HK-2 cells than on L-02 cells, and DON was less cytotoxic on C28/I2 cells than on L-02 and HK-2 cells (Table 1). T-2 toxin was also much more efficient inhibitor of proliferation than DON (Table 1).

**Table 1:** The IC50 values for T-2 toxin and DON on C28/I2, L-02 and HK-2 cells.

Mycotoxin	Time (h)	IC50 (ng/ml)		
		C28/I2	L-02	HK-2
T-2 toxin	24	36.84 ± 7.17^a^	17.51 ± 8.57^a^	72.76 ± 51.31^a^
	48	22.10 ± 9.85^a^	9.91 ± 5.95^b^	16.82 ± 4.99^ab^
	72	9.17 ± 4.12^a^	2.42 ± 1.38^b^	8.68 ± 1.56^a^
DON	24	1991.06 ± 159.83^a^	1095.77 ± 113.16^b^	1764.72 ± 493.28^a^
	48	1168.52 ± 224.36^a^	740.96 ± 205.71^b^	412.34 ± 155.52^b^
	72	751.45 ± 179.80^a^	439.46 ± 137.19^b^	194.98 ± 79.36^b^

IC50 is the dose required to inhibit cell proliferation by 50%. Results are expressed as mean ± SD (n = 3). Data in the same exposure time marked with different letters means significant difference (p < 0.05).

### Isobologram analysis

Isobologram analysis was used to determine the type of interaction between T-2 toxin and DON on C28/I2, L-02 and HK-2 cells. The values for parameters Dm, m, and r, of the combined T-2 toxin and DON, as well as CI values are presented for C28/I2 (Table 2), L-02 (Table 3) and HK-2 cells (Table 4). The IC10, IC25, IC50, IC75 and IC90 were the doses required to inhibit proliferation by 10%, 25%, 50%, 75% and 90%, respectively (Tables 2–4). The combinations of T-2 toxin and DON showed antagonistic effects on C28/I2 cells (CI = 1.59–2.60) and L-02 cells (CI = 1.30–4.50) at all times and deses tested (Tables 2 and 3). On HK-2 cells, the effects of T-2 toxin/DON combinations were antagonistic (CI = 1.13–3.41) after 24 and 48 h of exposure at all doses tested, but synergistic (CI = 0.83) at IC10 and antagonistic (CI = 1.01–2.36) at IC25 to IC90 after 72 h of exposure (Table 4).

**Table 2:** Dose–effect relationship parameters and mean combination index (CI) values of T-2 toxin or DON alone or in combination on C28/I2 cells.

Mycotoxin	Time (h)	Dose–effect parameters			CI values at the following effect levels				
		D_m_	m	r	IC10	IC25	IC50	IC75	IC90
T-2 toxin	24	38.52	0.65	0.9484					
	48	21.29	0.64	0.9529					
	72	8.73	0.82	0.9283					
DON	24	2142.89	0.91	0.9722					
	48	1202.48	1.03	0.9905					
	72	761.61	1.24	0.9722					
T-2 + DON	24	1847.50	0.78	0.9844	2.60 (Ant)	2.23 (Ant)	2.01 (Ant)	1.92 (Ant)	1.94 (Ant)
	48	816.32	0.79	0.9913	2.17 (Ant)	1.78 (Ant)	1.60 (Ant)	1.59 (Ant)	1.75 (Ant)
	72	471.09	0.89	0.9849	1.92 (Ant)	1.88 (Ant)	1.92 (Ant)	2.05 (Ant)	2.29 (Ant)

The parameters m, D_m_, and r are the slope, antilog of the x-intercept, and the linear correlation coefficient of the median-effect plot, which signifies the shape of the dose-effect curve, the potency (IC50), and the conformity of the data to the mass-action law, respectively. IC10, IC25, IC50, IC75, and IC90 are the doses required to inhibit proliferation by 10%, 25%, 50%, 75% and 90%, respectively. Ant indicates antagonistic effect.

**Table 3:** Dose–effect relationship parameters and mean combination index (CI) values of T-2 toxin or DON alone or in combination on L-02 cells.

Mycotoxin	Time (h)	Dose–effect parameters			CI values at the following effect levels				
		D_m_	m	r	IC10	IC25	IC50	IC75	IC90
T-2 toxin	24	16.49	0.60	0.9180					
	48	8.60	0.49	0.9400					
	72	2.11	0.63	0.9706					
DON	24	1194.16	1.22	0.9510					
	48	728.91	1.03	0.9757					
	72	426.62	1.12	0.9555					
T-2 + DON	24	785.86	0.66	0.9538	1.76 (Ant)	1.67 (Ant)	1.80 (Ant)	2.37 (Ant)	3.81 (Ant)
	48	352.80	0.52	0.9600	1.35 (Ant)	1.30 (Ant)	1.47 (Ant)	2.21 (Ant)	4.50 (Ant)
	72	123.01	0.63	0.9706	2.41 (Ant)	1.96 (Ant)	1.70 (Ant)	1.70 (Ant)	2.13 (Ant)

The parameters m, D_m_, and r are the slope, antilog of the x-intercept, and the linear correlation coefficient of the median-effect plot, which signifies the shape of the dose-effect curve, the potency (IC50), and the conformity of the data to the mass-action law, respectively. IC10, IC25, IC50, IC75, and IC90 are the doses required to inhibit proliferation by 10%, 25%, 50%, 75% and 90%, respectively. Ant indicates antagonistic effect.

**Table 4:** Dose–effect relationship parameters and mean combination index (CI) values of T-2 toxin or DON alone or in combination on HK-2 cells.

Mycotoxin	Time (h)	Dose–effect parameters			CI values at the following effect levels				
		D_m_	m	r	IC10	IC25	IC50	IC75	IC90
T-2 toxin	24	55.48	0.80	0.9921					
	48	16.19	0.80	0.9802					
	72	8.57	1.04	0.9472					
DON	24	1754.26	0.61	0.9463					
	48	381.38	0.56	0.9666					
	72	183.31	0.74	0.9753					
T-2 + DON	24	1623.01	0.63	0.9906	1.37 (Ant)	1.46 (Ant)	1.62 (Ant)	1.88 (Ant)	2.28 (Ant)
	48	412.02	0.54	0.9643	1.13 (Ant)	1.33 (Ant)	1.67 (Ant)	2.29 (Ant)	3.41 (Ant)
	72	157.68	0.69	0.9451	0.83 (Syn)	1.01 (Ant)	1.29 (Ant)	1.71 (Ant)	2.36 (Ant)

The parameters m, D_m_, and r are the slope, antilog of the x-intercept, and the linear correlation coefficient of the median-effect plot, which signifies the shape of the dose-effect curve, the potency (IC50), and the conformity of the data to the mass-action law, respectively. IC10, IC25, IC50, IC75, and IC90 are the doses required to inhibit proliferation by 10%, 25%, 50%, 75% and 90%, respectively. Ant and Syn indicate antagonistic and synergistic effect, respectively.

### Cell ultrastructure changes induced by T-2 toxin and/or DON

**Figure 4:**
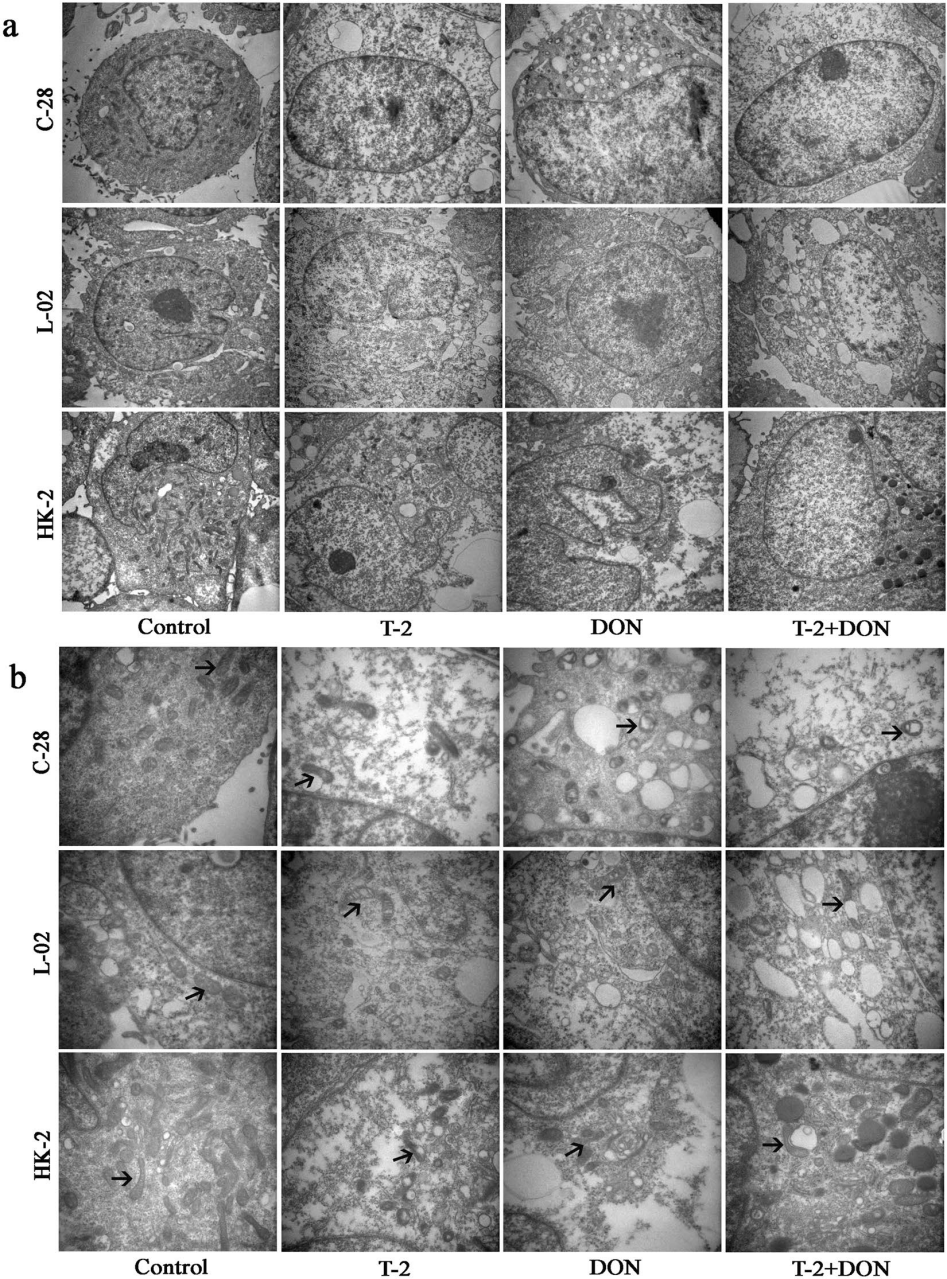
Ultrastructural changes induced by T-2 toxin and/or DON. C28/I2, L-02 and HK-2 cells were exposed to T-2 toxin (10 ng/ml) or DON (800 ng/ml), alone or in combination for 24 h and visualized by transmission electron microscopy (TEM) (a. TEM × 10,000; b. TEM × 30,000). Arrows indicate mitochondria.

To visualize morphological changes in C28/I2, L-02, and HK-2 cells after exposure to mycotoxins for 24 h, the cellular ultramicroscopic structures were view by TEM. Compared to untreated control cells, the electron density in nucleus and cytoplasm of each cell line decreased to a different extent (Fig. 4a), and swelling, vacuolar degeneration, and increased density of mitochondria were also observed after exposure all three cell lines to T-2 toxin or DON alone or in combination (Fig. 4b).

### Cell cycle progression induced by T-2 toxin and/or DON

**Figure 5:**
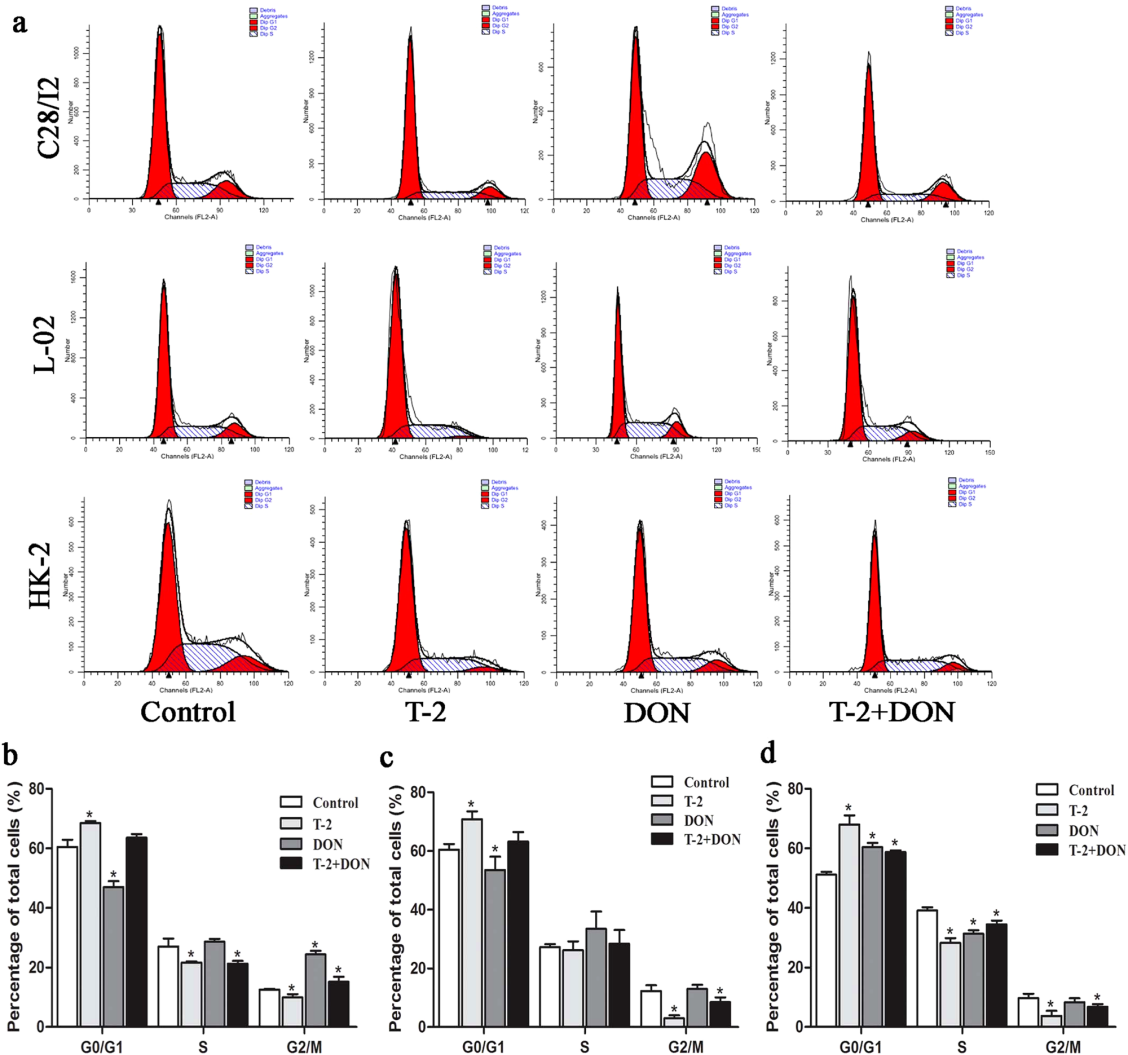
Cell cycle arrest induced by T-2 toxin and/or DON. (**a**) C28/I2, L-02, and HK-2 cells were exposed to T-2 toxin (10 ng/ml) or DON (800 ng/ml), alone or in combination, for 24 h. and cell cycle phase distribution was analyzed by flow cytometry. Cell cycle phase distribution was quantified as the percentage of total cells in C28/I2 (**b**), L-02 (**c**), and HK-2 (**d**) cells. Data are represented as the mean ± SD from three independent experiments; *P < 0.05 indicates significance versus untreated control values.

Flow cytometry analysis of cell cycle progression after exposure to T-2 toxin or, DON alone or in combination for 24 h is shown in Fig. 5a. For C28/I2 cells, T-2 toxin significantly induced accumulation of cells in the G0/G1 phase, while DON alone or in combination with T2-toxin significantly induced accumulation of cells in the G2/M phase when compared to untreated control cells (Fig. 5b). For L-02 cells, T-2 toxin significantly induced accumulation of cells in the G0/G1 phase, but cell cycle arrest in the presence of DON alone or together with T2-toxin was not significantly from that in untreated control cells (Fig. 5c). For HK-2 cells, T-2 toxin and/or DON caused a significant arrest in the G0/G1 phase, which was accompanied by decreased accumulation of cells in the S and G2/M phase when compared to untreated control cells (Fig. 5d).

### Apoptosis induced by T-2 toxin and/or DON

**Figure 6:**
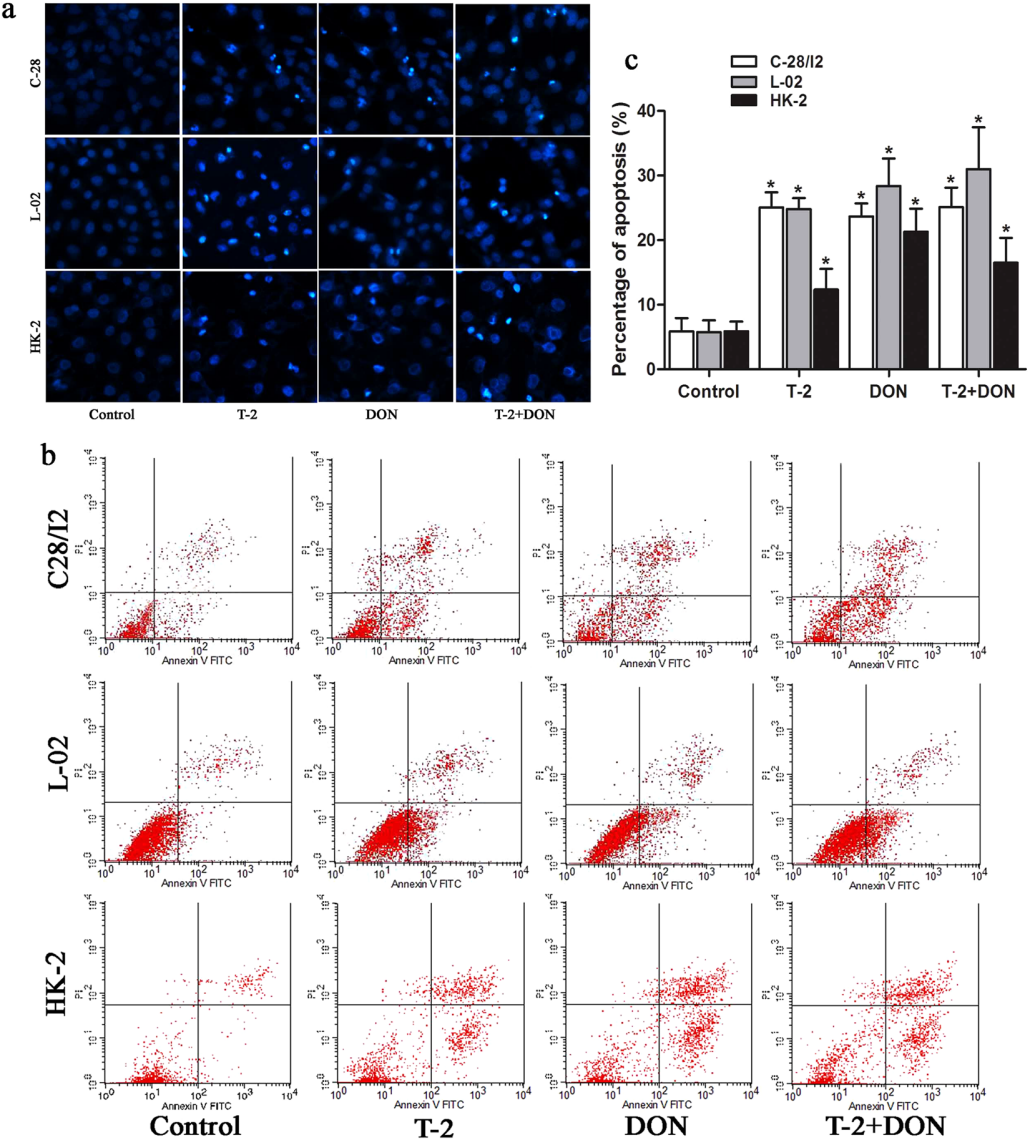
Apoptosis induced by T-2 toxin and/or DON. C28/I2, L-02, or HK-2 cells were exposed to T-2 toxin (10 ng/ml) or DON (800 ng/ml), alone or in combination for 24 h and analyzed for (**a**) morphologic changes of apoptotic cells by staining with Hoechst 33342, visualized by a fluorescence microscopy (Magnification: 200×) and (**b**) apoptotic rate by flow cytometry; (**c**) apoptotic rate was quantified as the percentage of apoptotic cells. Data are represented as the mean ± SD from three independent experiments; *P < 0.05 indicated significant differences versus untreated control values.

Changes in the nuclei of C28/I2, L-02, and HK-2 cells due to T-2 toxin and/or DON exposure were studied by Hoechst 33342 staining. All three cell lines treated with T-2 toxin or DON alone or in combination for 24 h exhibited typical morphologic changes of apoptotic cells, with the appearance of irregularly shaped nuclei and fragmented chromatin (Fig. 6a). Flow cytometry analysis showed that the proportions of apoptotic cells were significantly increased when cells exposed to mycotoxins for 24 h compared to untreated cells (Fig. 6b and c). HK-2 cells were most resistant against apoptosis.

### Oxidative stress induced by T-2 toxin and/or DON

**Figure 7:**
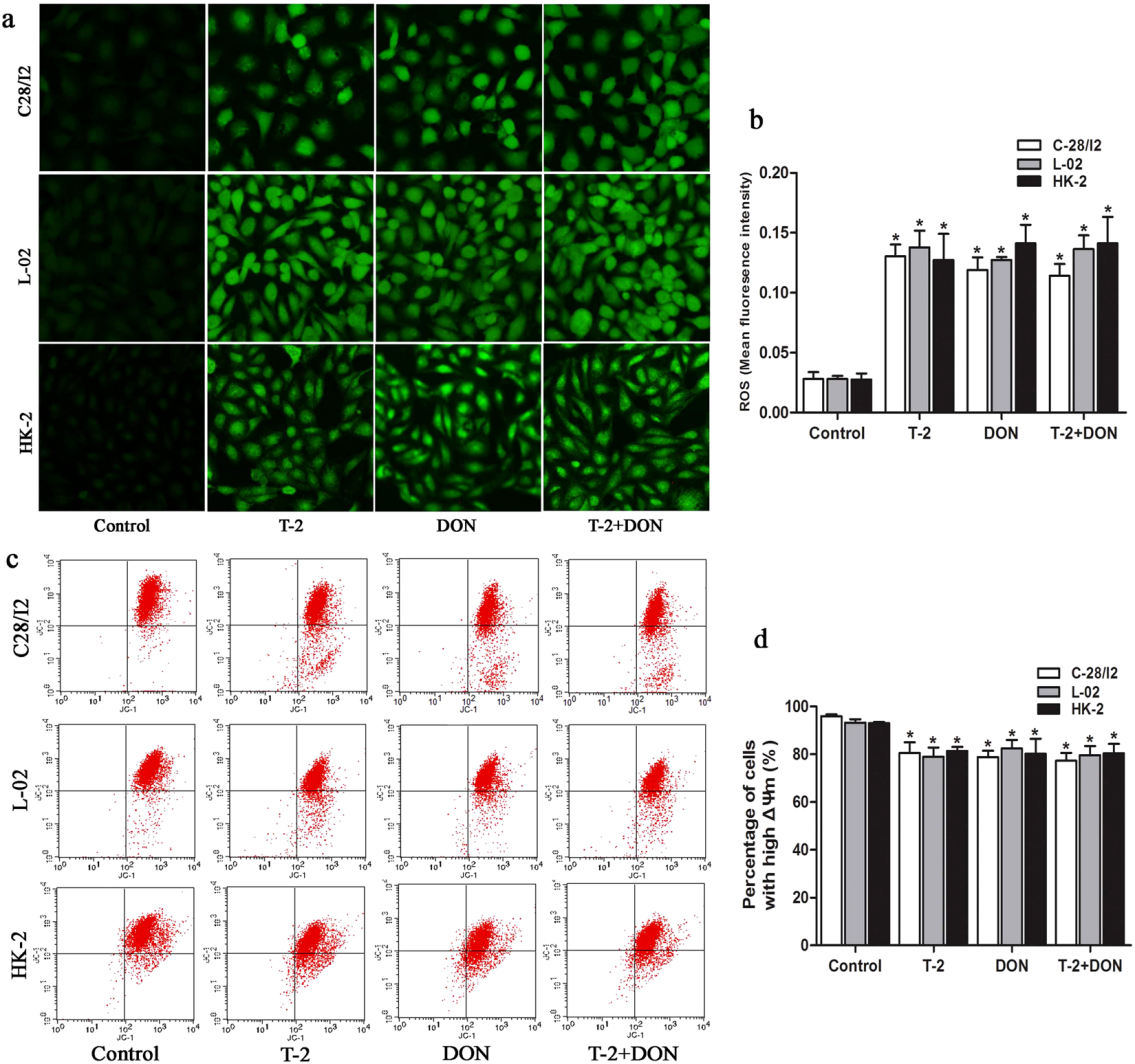
Oxidative stress and mitochondrial membrane potential induced by T-2 toxin and/or DON. C28/I2, L-02 and HK-2 cells were exposed to T-2 toxin (10 ng/ml) or DON (800 ng/ml), alone or in combination, for 24 h. (**a**) ROS was visualized in cell layers by fluorescence microscopy (Magnification: 200×) and (**b**) quantified as ROS fluorescence intensity. (**c**) The changes in ΔΨm were analyzed by flow cytometry and (**d**) quantified as the percentage of cells with high ΔΨm. Data are represented as the mean ± SD from three independent experiments; *P < 0.05 indicates significant differences versus untreated control values.

Oxidative stress is involved in the apoptosis induced by mycotoxins. In the present study, we found that the ROS production in C28/I2, L-02, and HK-2 cells was significantly increased by mycotoxin treatment for 24 h compared to untreated controls (Fig. 7a and b).

### Mitochondrial membrane potential induced by T-2 toxin and/or DON

ROS accumulation induces mitochondrial permeability transition and mitochondrial membrane potential (ΔΨm) loss. The changes in ΔΨm induced by T-2 toxin and/or DON were detected with a fluorescent dye JC-1, which gives a red fluorescence when ΔΨm is high and green fluorescence when ΔΨm is low. C28/I2, L-02 and HK-2 cells treated with mycotoxin for 24 h all displayed loss of ΔΨm with significantly decreased percentages of cells with high ΔΨm (Fig. 7c and d).

### The distribution of T-2 toxin and DON in knee joint, liver and kidney

**Figure 8:**
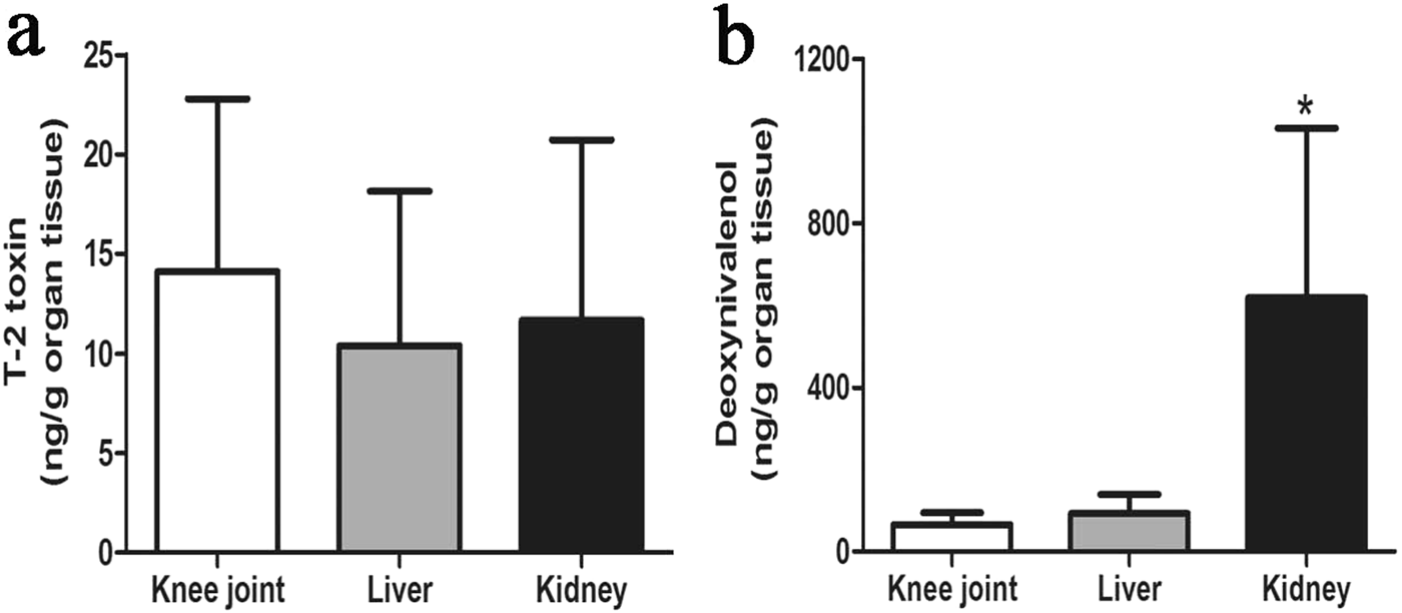
The distribution of mycotoxins in the knee joint, liver, and kidney. Sprague-Dawley rats were administered a single dose of (**a**) T-2 toxin at 2 mg/kg bw or (**b**) DON at 10 mg/kg bw by oral gavage and tissues were harvested after 8 h analyzed for mycotoxin concentrations. Data are represented as mean ± SD (n = 6); *P < 0.05 indicates significant difference in DON concentration in the kidney versus knee joint and liver groups.

The recovery rates of T-2 toxin and DON from tissues were 66%~114% and 65%~115%, respectively. Following 8 h of the acute contamination of SD rats, the concentrations of T-2 toxin were 14.34 ± 8.69, 10.41 ± 7.74, 11.66 ± 9.07 ng/g of organ tissue in knee joint, liver and kidney, respectively. The differences were not significant among the three tissues (Fig. 8a). Meanwhile, the concentrations of DON were 66.22 ± 28.64, 92.96 ± 46.14, 616.42 ± 415.31 ng/g organ tissue in knee joint, liver and kidney, respectively. Notably, the concentrations in knee joint and liver were significantly lower than that in kidney (Fig. 8b).

## Discussion

Grain contamination with mycotoxins has been convincingly associated with KBD in the past decades. However, contamination with mycotoxins in food is a common problem existing in many countries. Whether KBD is simply an environmentally related disease or directly caused by mycotoxins remains controversial because KBD is found only in a limited regions, including Northeast to Southwest China, Southeastern Siberia, and North Korea^13, 14^. The present study compared the effects of individual and combined treatments with T-2 toxin and DON using three cell lines derived from kidney, liver, and cartilage and suggested that the damaging effects mycotoxins on cartilage in KBD are not specific to chondrocytes.

The toxic effects of T-2 toxin and DON on cartilage are well established and previous studies have demonstrated the possible relationship between these mycotoxins and the risk of KBD^37, 38^. Cartilage is the main anatomical sites affected by KBD in patients. However, a previous study showed that the effects of fusarochromanone and T-2 toxin on articular chondrocytes were not specific^39^. The results of our study also showed that T-2 toxin or DON, alone or in combination, could inhibit cell proliferation of cell lines derived from cartilage (C28/I2), liver (L-02), and kidney (HK-2) cells in a time- and dose-dependent manner. This suggests that the damaging effects of T-2 toxin and DON are not specific for chondrocytes. In addition, the cytotoxicity of T-2 toxin and DON on L-02 and HK-2 cells were greater than on chondrocytes, which suggests that liver and kidney cells may be more sensitive than chondrocytes to these mycotoxin. Morphological examination showed decreased electron density in the nucleus and cytoplasm and altered mitochondria in the three in all three cell lines after T-2 toxin and/or DON exposure.

Mycotoxins coexist naturally in feed and food worldwide. Thus, it is necessary to take into account the toxicity of mycotoxin mixtures. The cytotoxicity of T-2 toxin and DON alone or in combination, has been investigated in Chinese hamster ovary (CHO-K1) and mammalian kidney epithelial (Vero) cells, and antagonistic effects were observed^10, 11^. In the present study, T-2 toxin and DON combinations showed antagonistic effects on C28/I2, L-02 and HK-2 cells at all times of exposure tested, there was synergism at a lower doses on HK-2 cells. The results indicate that mixtures of T-2 toxin and DON exert similar antagonistic interaction effects on the three cultured cells.

Apoptosis is a form of programmed cell death that occurs in aging or damaged cells^40^. T-2 toxin and DON have been shown to induce apoptosis in many cell types^41^. Apoptosis has been assumed to be a crucial pathological change in chondrocytes of KBD patients, and recent studies have indeed shown that T-2 toxin induced apoptosis in human chondrocytes. This finding suggests a relationship between T-2 toxin and risk of KBD^32, 42^. However, apoptosis is not the primary pathological feature in chondrocytes of KBD patients, and apoptotic chondrocytes and similar changes in expression of apoptosis-related genes were observed in the cartilage from patients with primary osteoarthritis^43^. Based on the results of our present study, T-2 toxin and DON individually or together can induce apoptosis in cell lines derived from tissues other than cartilage, suggesting that apoptosis induced by these mycotoxins is not specific to human chondrocytes.

Cell cycle arrest is one of the most important toxic effects of many mycotoxins. Previous studies showed that T-2 toxin induced G0/G1 phase arrest in differentiated murine embryonic stem cells^7^, and DON induced G2/M phase arrest in human umbilical vein endothelial cells and human epithelial cells^44, 45^. Our study showed that T-2 toxin resulted in G0/G1 phase arrest in C28/I2, L-02 and HK-2 cells, while significant G2/M phase arrest induced by DON was observed only in C28/I2 cells. However, the limitation of this data is that we analysed cell cycle only using a single dose for 24 h.

Several studies have shown that oxidative stress is involved in the apoptosis induced by T-2 toxin and DON^41^. Our study showed that T-2 toxin or DON, either individually or combined, can significantly increase the ROS generation in C28/I2, L-02, and HK-2 cells. It is widely recognized that the induction of ROS can modulate the mitochondrial membrane potential, triggering the release of cytochrome c into the cytoplasm to initiate the apoptotic pathway^46, 47^. Moreover, many reports have shown that T-2 toxin and DON induce apoptosis through ROS-mediated mitochondrial pathway in different cell types^7, 48–50^. In the present study, we observed swelling, vacuolar degeneration and increased density of mitochondria in all three cell types after treatment with T-2 toxin and/or DON. In addition, a simultaneous decrease of mitochondrial membrane potential was also observed. These results suggest that ROS-mediated mitochondrial pathway was possibly involved in the T-2 toxin and/or DON-induced apoptosis of C28/I2, L-02 and HK-2 cells, but it need to be further investigated when blocking of ROS accumulation.

T-2 toxin and DON are rapidly absorbed and distributed in animal tissues after exposure. Previous studies showed that concentrations of T-2 toxin and DON in plasma and most tissues in mice peaked within 15 min following oral administration, and then rapidly declined in a biphasic pattern^36, 51^. In the present study, we analyzed the concentrations of T-2 toxin and DON in the knee joint, liver, and kidney of SD rats at 8 h after administration with a single dose of these two mycotoxins based on previous studies^36, 52, 53^, with consideration of the absorption and clearance features of these two toxins in animal bodies after exposure, and the sensitivity of the ELISA kits used in this study. The study showed that the concentrations of T-2 toxin in the knee joint, liver, and kidney were not significantly different, but the concentrations of DON in knee joint and liver were significantly lower than in the kidney. Our results suggest that T-2 toxin and DON do not preferentially accumulate in joint tissues.

In conclusion, using cell lines derived from cartilage, liver, and kidney as models, we found that treatment with T-2 toxin and/or DON showed time- and concentration-dependent cytotoxic effects on cell viability. The combination of T-2 toxin and DON exhibited a similar antagonistic effect. Exposure to T-2 toxin or DON, alone or in combination could also induce cell ultrastructural changes, especially in the cytoplasm and mitochondria, increased oxidative stress with ROS generation, and decreased mitochondrial membrane potential, accompanied by cell cycle arrest and increased apoptosis in all three culture models of C28/I2, L-02 and HK-2 cell lines. In addition, T-2 toxin and DON accumulate in joint tissues to a similar extent as they do in liver and kidney. Our findings suggest, therefore, that the damaging effects of T-2 toxin and/or DON on chondrocytes were not specific *in vitro*. Whether T-2 toxin and DON contribute directly to the etiology of KBD and why these mycotoxins selectively damage the cartilage in KBD patients will deserve further study.
